# *MAPT* haplotype-associated transcriptomic changes in progressive supranuclear palsy

**DOI:** 10.1186/s40478-024-01839-3

**Published:** 2024-08-17

**Authors:** Hadley W. Ressler, Jack Humphrey, Ricardo A. Vialle, Bergan Babrowicz, Shrishtee Kandoi, Towfique Raj, Dennis W. Dickson, Nilüfer Ertekin-Taner, John F. Crary, Kurt Farrell

**Affiliations:** 1 Department of Pathology, Icahn School of Medicine at Mount Sinai, 1 Gustave L. Levy Place Box 1194, New York, NY 10029 USA; 2 Department of Artificial Intelligence and Human Health, Icahn School of Medicine at Mount Sinai, New York, NY USA; 3 Nash Family Department of Neuroscience, Icahn School of Medicine at Mount Sinai, New York, NY USA; 4 Ronald M. Loeb Center for Alzheimer’s Disease, Icahn School of Medicine at Mount Sinai, New York, NY USA; 5 Friedman Brain Institute, Icahn School of Medicine at Mount Sinai, New York, NY USA; 6 Neuropathology Brain Bank and Research CoRE, Icahn School of Medicine at Mount Sinai, New York, NY USA; 7 Department of Genetics and Genomic Sciences and Icahn Institute for Data Science and Genomic Technology, Icahn School of Medicine at Mount Sinai, New York, NY USA; 8 Estelle and Daniel Maggin Department of Neurology, Icahn School of Medicine at Mount Sinai, New York, NY USA; 9 Department of Neuroscience, Mayo Clinic, Jacksonville, FL USA

**Keywords:** Tauopathy, Progressive supranuclear palsy, RNA-seq, 17q21.31, *MAPT* haplotype, *KANSL1*

## Abstract

Progressive supranuclear palsy (PSP) is a neurodegenerative movement and cognitive disorder characterized by abnormal accumulation of the microtubule-associated protein tau in the brain. Biochemically, inclusions in PSP are enriched for tau proteoforms with four microtubule-binding domain repeats (4R), an isoform that arises from alternative tau pre-mRNA splicing. While preferential aggregation and reduced degradation of 4R tau protein is thought to play a role in inclusion formation and toxicity, an alternative hypothesis is that altered expression of tau mRNA isoforms plays a causal role. This stems from the observation that PSP is associated with common variation in the tau gene (*MAPT*) at the 17q21.31 locus which contains low copy number repeats flanking a large recurrent genomic inversion. The complex genomic structural changes at the locus give rise to two dominant haplotypes, termed H1 and H2, that have the potential to markedly influence gene expression. Here, we explored haplotype-dependent differences in gene expression using a bulk RNA-seq dataset derived from human post-mortem brain tissue from PSP (*n* = 84) and controls (*n* = 77) using a rigorous computational pipeline, including alternative pre-mRNA splicing. We found 3579 differentially expressed genes in the temporal cortex and 10,011 in the cerebellum. We also found 7214 differential splicing events in the temporal cortex and 18,802 in the cerebellum. In the cerebellum, total tau mRNA levels and the proportion of transcripts encoding 4R tau were significantly increased in PSP compared to controls. In the temporal cortex, the proportion of reads that expressed 4R tau was increased in cases compared to controls. 4R tau mRNA levels were significantly associated with the H1 haplotype in the temporal cortex. Further, we observed a marked haplotype-dependent difference in *KANSL1* expression that was strongly associated with H1 in both brain regions. These findings support the hypothesis that sporadic PSP is associated with haplotype-dependent increases in 4R tau mRNA that might play a causal role in this disorder.

**Supplementary Information:**

The online version contains supplementary material available at 10.1186/s40478-024-01839-3.

## Introduction

The tau proteinopathies (“tauopathies”) are a group of neurodegenerative disorders characterized neuropathologically by intracellular accumulation of the microtubule-associated protein tau in the brain [1]. The tauopathies have broad clinical heterogeneity and overlap, variably displaying movement disorder, cognitive impairment, motor neuron disease, and psychiatric symptomatology. Thus, obtaining a better understanding of the complex mechanisms that underlie tau accumulation and toxicity has the potential to enable better diagnostic and therapeutic strategies for many patients [2, 3]. Genetic factors play a role, with some kindreds of frontotemporal lobar degeneration (FTLD) harboring autosomal dominant mutations in the tau gene (*MAPT*) that involve coding regions and splice sites, demonstrating that primary involvement of tau is sufficient to cause neurodegeneration [4–7]. These mutations, which preferentially cluster around exon 10, influence tau pre-mRNA alternative splicing leading to an imbalance of tau proteoform expression [8]. However, the majority of tauopathy cases are sporadic without a family history or autosomal dominant mutation.

Progressive supranuclear palsy (PSP), a dementing movement disorder, is the most common primary sporadic tauopathy [1]. It is characterized neuropathologically by preferential accumulation of tau protein with 4-repeat binding domains (4R tau) in the basal ganglia, brainstem, and cerebellum [9, 10]. The relative abundance of tau isoforms is dictated by alternative pre-mRNA splicing of *MAPT* exon 10, which encodes the second of the four tandem microtubule-binding domain repeats (which leads to increased binding affinity) [11–14]. While sporadic PSP patients do not have *MAPT* mutations, such as those in and around exon 10 splice sites that alter 4R tau mRNA, it is associated with common genetic variation [15–17]. *MAPT* is situated within the highly complex 17q21.31 locus with numerous studies delineating an approximately one megabase inversion region flanked by large insertion-deletion polymorphisms giving rise to the H1 and H2 haplotypes as well as numerous subhaplotypes. H1 is the more common haplotype and has been found to be significantly associated with PSP [18–22]. While *MAPT* is roughly at the center of the inversion region, the base pair sequence of the six tau isoforms are not predicted to be directly altered by this structural variation. In contrast, both haplotypes contain regions that result in partial duplication of the neighboring *KANSL1* gene. The H1 haplotype contains a polymorphic β duplication which affects *KANSL1*, *ARL17*, and *LRRC37A*, while the H2 haplotype contains a shorter α duplication region within β which includes only the 5’ exons of *KANSL1* [19, 23, 24]*.* These structural changes are predicted to give rise to truncated pseudogenes, but these are poorly studied and the extent to which they are transcribed and translated to functional polypeptides remains unclear [25]. These structural variants have the potential to lead to dramatic changes in gene expression in the locus that could participate in the pathogenesis of PSP.

While PSP is characterized by preferential accumulation of 4R tau protein, it has yet to be established whether total tau and the 4R tau isoform mRNA are differentially transcribed and translated [9, 10, 26–28]. Chambers et al. found increased levels of 4R tau mRNA in the brainstem but not in cerebellum or cortex of patients with PSP, while Takanashi et al. did find increased 4R tau mRNA in the frontal cortex and globus pallidus of patients with PSP [10, 29]. One study found increased 4R tau mRNA in the frontal cortex compared to 3R tau, but found lower overall tau mRNA levels in PSP compared to controls [30]. Another study found upregulated total tau expression specifically in the astrocytes of patients with PSP [31]. Further studies considered the association between 4R tau mRNA and the *MAPT* H1 and H2 haplotypes, with mixed observations. Some studies have found increased total tau expression in the H1 haplotype [22, 32, 33], while another study found no association between tau expression and the H1 haplotype [30]. Additionally, it has been suggested that the association between tau expression and the H1 haplotype is due to a technical artifact, with differences in hybridization affinity secondary to imperfect matches between probes between H1 and H2 [34, 35]. Thus, the extent to which there are haplotype-dependent differences in tau mRNA expression remains controversial, as is the association between PSP and 4R tau mRNA in various brain regions.

In this study, we sought to further test the hypothesis that increased levels of total tau and 4R tau mRNA in post-mortem human brain tissue are associated with PSP, and if there are differences based on regional vulnerability. We also sought to examine whether other genes at the 17q21.31 locus are differentially expressed or spliced which may contribute to pathology. To accomplish this, we leveraged a streamlined computational pipeline, including differential intron excision, to reanalyze an existing RNA-seq dataset from post-mortem brain tissues from a large collection of autopsy-confirmed PSP cases and controls. Genotyping of tagged single nucleotide polymorphisms (SNPs) allowed us to correlate *MAPT* haplotypes with the expression of candidate genes and isoforms.

## Methods

### Dataset

The progressive supranuclear palsy (PSP) RNA-seq data (Synapse ID: SYN3163039) was obtained from synapse.org and was derived from the cerebellum and temporal cortex from a total of 164 neuropathologically confirmed cases (*n* = 84) and controls (*n* = 80) before quality control (QC) [36]. All 164 data were processed through our pipeline. Tau haplotype was derived from preexisting corresponding genome-wide association study data obtained from NIAGADS (NG00037) using the tagging SNP rs1800547 as previously described [37]. Genomic annotations for α, β, and γ regions within the haplotype were lifted from hg19 to hg38 using the UCSC Genome Browser [19]. All the data was generated at the Mayo Clinic Jacksonville.

### RNA-seq processing pipeline

We used the RAPiD-nf pipeline developed as part of the CommonMind consortium [38]. RAPiD-nf is a pipeline in the NextFlow framework and uses Trimmomatic (version 0.36) [39], STAR (version 2.7a) [40], FASTQC (version 0.11.8) [41], featureCounts (version 1.3.1) [42], and Picard (version 2.20.0) [43] for pre-processing and quality control. RSEM (version 1.3.1) [44] was used for differential gene expression, and LeafCutter (version 0.2.8) [45] was used for differential intron excision. After all QC steps, 3 samples were excluded from the temporal cortex, and 4 were excluded from the cerebellum.

### Covariate adjustment

The gene expression matrix was normalized using trimmed mean of M values and transformed using the limma::voom() function [46]. Lowly expressed genes were removed. Covariates were selected to minimize gene expression differences based on technical and biological variables. Clinical and technical variables from Picard were combined and correlated using variancePartition [47]. Variables that contributed the most to variance in gene expression and had the least overlap with one another were included. The final variables included as covariates were RNA integrity number (RIN), percent duplicate reads, mean insert size, age, sex, and case–control status. Of note, post-mortem interval (PMI) was not considered as a covariate due to insufficient data availability.

### Differential gene expression

After normalization and covariate adjustment, differential gene expression (DGE) analysis was performed using the limma package to compare gene expression of PSP cases and controls [48]. Limma calculated log2-fold change, t statistics, and *p* values for each gene. Statistics were computed using the *treat* method which calculates *p* values from empirical Bayes moderated *t*-statistics with a minimum log2 fold change (logFC) requirement [46].

### Pathway analysis

We used Ingenuity Pathway Analysis (IPA) software (Qiagen, Hilden Germany) to investigate whether the differentially expressed genes were enriched in certain biological processes and pathways. The IPA Core Analysis module was employed to analyze the differentially expressed genes (*p* < 0.05) against the Ingenuity Knowledge Base (Genes Only) reference set. The analysis produced a list of enriched pathways and processes with corresponding *p* values and z-scores, and the most significant ones were identified based on these statistical measures.

### Differential splicing

We used the LeafCutter package in R with Regtools (version 0.5.2) for differential intron excision analysis to examine splicing [45]. BAM files were preprocessed to generate junction and intron files. Next, Leafcutter was employed to perform the differential splicing analysis. The outputs were processed and visualized using LeafViz (github.com/jackhump/leafviz). To focus on statistically significant intron–exon junction regions, only those with a Bayes factor greater than 10 and a false discovery rate (FDR) less than 0.05 were included in the results. Percent exon inclusion for *MAPT* exon 10 was calculated as mean(flanking introns %)/(mean(flanking introns %) + skipping intron %). To assess the association between *MAPT* and *KANSL1* isoforms detected through LeafCutter and *MAPT* haplotype status, an ANOVA was performed which compared expression of each isoform with sample haplotype status (homozygous H1, heterozygous H1H2, and homozygous H2H2).

### Deconvolution analysis

To perform gene expression deconvolution analysis, we used BRETIGEA (BRain cEll Type specIfic Gene Expression Analysis) [49]. The raw gene expression count matrix was input to BRETIGEA to estimate the proportions of neuronal and glial cell types present in the samples. These cell-type proportions were used to estimate the cellular composition of the samples but were not incorporated into the gene expression matrix due to lack of ability to validate the cell type proportions.

## Results

We leveraged a publicly available bulk RNA-sequencing dataset from post-mortem brain tissue from the cerebellum and temporal cortex [36]. After quality control, the dataset consisted of 84 PSP cases and 77 controls (Table 1). Of these, there were 51 male and 33 female PSP cases that were compared to 38 male and 39 female controls. The average age of temporal cortex controls was 82.3 ± 1.0 (53–90) and the average age of temporal cortex PSP cases was 73.9 ± 0.7 (61–89) (*p* < 0.0001); the average age of cerebellum controls was 79.6 ± 0.8 (58–89) and the average age of cerebellum PSP cases was 74.1 ± 0.7 (61–89) (*p* < 0.0001). The average RNA integrity number (RIN) of temporal cortex controls was 7.6 and the average RIN of temporal cortex PSP cases was 8.5 (*p* < 0.0001); the average RIN of cerebellum controls was 7.7; the average RIN of cerebellum PSP cases was 8.5 (*p* < 0.0001). The average post-mortem interval (PMI) of temporal cortex controls was 6.2 h and the average PMI of temporal cortex PSP cases was 8.6 h (*p* = 0.09); The average PMI of cerebellum controls was 6.2 h and the average PMI of cerebellum PSP cases was 8.3 h (*p* = 0.14).

**Table 1:** Clinical data

Diagnosis	Cerebellum					Temporal cortex				
	n	M/F (n)	Average age (yr ± SEM, range)	RIN	PMI	n	M/F (n)	Average age (yr ± SEM, range)	RIN	PMI
PSP	83	50/33	74.1 ± 0.7 (61–89)	8.5	8.3	84	51/33	73.9 ± 0.7 (61–89)	8.5	8.6
Control	77	40/37	79.6 ± 0.8 (58–89)	7.7	6.2	77	38/39	82.3 ± 1.0 (53–90)	7.6	6.2
Total	160	90/70	76.7 ± 0.6 (58–89)	8.1	7.3	161	89/72	77.9 ± 0.7 (53–90)	8	7.9
*p* value			< 0.0001	< 0.0001	0.14			< 0.0001	< 0.0001	0.09

*RIN* RNA integrity score, *PMI* Post mortem interval

**Fig. 1:**
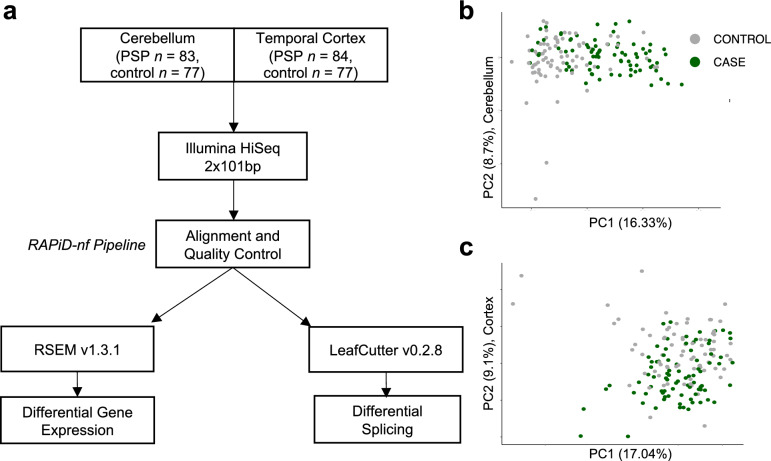
RNA sequencing pipeline using RAPiD-nf aligns and quality controls 161 samples for differential expression and splicing analysis. **a** RNA-seq samples were obtained from Synapse.org [50]. The cohort included 161 PSP cases and controls, with samples taken from the cerebellum and temporal cortex. Samples were processed using RAPiD-nf, a processing pipeline in the NextFlow framework. RAPiD-nf uses Trimmomatic, STAR, FASTQC, featureCounts, Pathogen, and Picard for pre-processing and quality control. RSEM and Limma were used for differential gene expression, and Regtools and LeafCutter were used for differential intron excision. **b**, **c** Principal component analysis of the RNA-seq expression matrix after covariate adjustment in cerebellum and temporal cortex shows no remaining outliers. 161 samples were included in downstream analysis

For alignment and quality control, we ran the RAPiD-nf pipeline which trims, aligns, quantifies gene expression, and performs quality control (Fig. 1). The mean read depth was 64.0 million reads for the cerebellum and 62.7 million for the temporal cortex. The mean percentage of reads that were aligned to the reference was 98.7% for the cerebellum and 98.2% for the temporal cortex. The mean percentage of reads mapping to mRNA in the samples was 71.5% for the cerebellum and 73.2% for the temporal cortex. The mean percentage ribosomal RNA was 3.1% for the cerebellum and 4.9% for the temporal cortex. Before filtering, 58,929 genes were included. After removing genes with no reads or a low read depth, 21,478 genes remained (Supplemental Fig. 1). Covariates were selected using variancePartition, and the final variables used for downstream analysis were RIN, percent duplicate reads, mean insert size, age, sex, and case–control status (Supplemental Fig. 2). Raw counts generated were used to estimate relative cell type proportions in each sample using BRETIGEA, which found that the cerebellum contained a different proportion of astrocytes, oligodendrocytes, and OPCs in cases compared to controls, and the temporal cortex contained a different proportion of neurons and oligodendrocytes in cases compared to controls (*p* < 0.05; Supplemental Fig. 3).

**Fig. 2:**
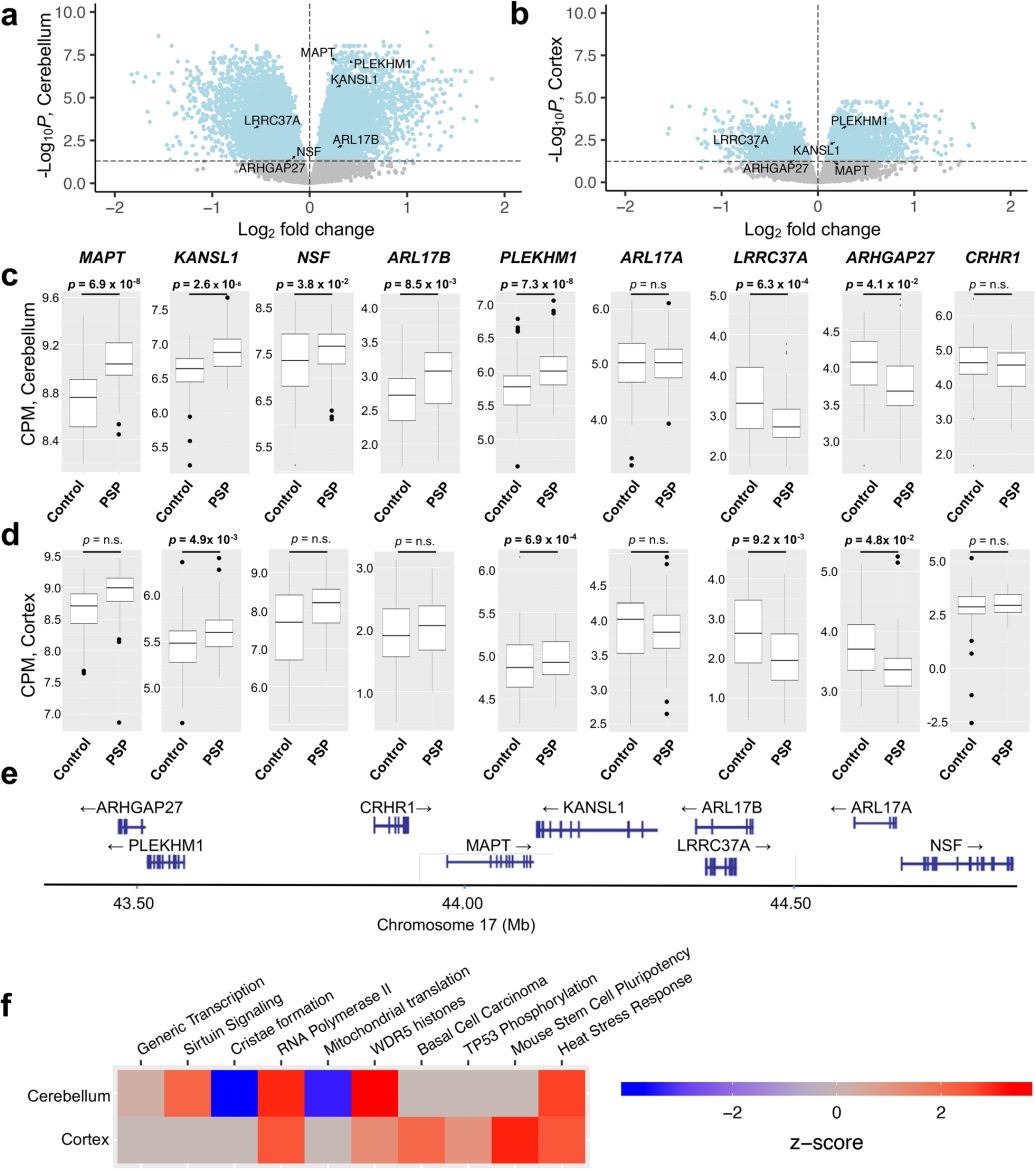
Genes within the 17q21.31 locus are differentially expressed between progressive supranuclear palsy cases and controls. 10,011 genes were differentially expressed in the cerebellum (**a**) and 3579 genes were differentially expressed in the temporal cortex (**b**; *p* < 0.05, FDR adjusted). (**c**, **d**) *MAPT*, *KANSL1*, *NSF, PLEKHM1, LRRC37A,* and *ARHGAP27* were differentially expressed in the cerebellum. *KANSL1, PLEKHM1, LRRC37A, and ARHGAP27* were differentially expressed in the temporal cortex. *ARL17A* and *CRHR1* were not differentially expressed in either brain region. **e** Map of the *MAPT* locus with location of genes examined. **f** IPA pathway analysis of top 5 canonical pathways from both the cerebellum and temporal cortex in order of p value, with z-score displayed

Using a significance threshold of *p* < 0.05 (FDR adjusted), 3579 genes were differentially expressed in the temporal cortex, with 2217 genes upregulated and 1362 genes downregulated in cases compared to controls (Fig. 2a, Supplemental Table 1). In the cerebellum, 10,011 genes were differentially expressed with 4870 genes upregulated and 5141 genes downregulated in cases compared to controls (Fig. 2b, Supplemental Table 2). Ingenuity pathway analysis (IPA) was used for pathway analysis for all cerebellum and temporal cortex DEGs (Supplemental Tables 3 and 4). The top signaling pathways included activation of the generic transcription, sirtuin, cristae formation, WDR5 histone modification, and tp53 phosphorylation pathways. The five signaling pathways with the lowest *p* values from each brain region are shown in Fig. 2e.

Given the established strong association of the 17q21.31 *MAPT* locus with PSP, we next focused on the genes within the region (Fig. 2c–e, Table 2). We found that in the cerebellum, a region vulnerable to degeneration in PSP, nearly every gene in the 17q21.31 locus was differentially expressed, including *MAPT, KANSL1, NSF, PLEKHM1, ARL17B, LRRC37A,* and *ARHGAP27.* The only genes at the locus that were not differentially expressed were *ARL17A* and *CRHR1*. In the temporal cortex, which is typically unaffected by PSP, there was differential expression of *KANSL1, PLEHKM1, LRRC37A,* and *ARGHAP27. MAPT* was not differentially expressed in this region but showed a trending increase in expression (*p* = 0.06, FDR adjusted).

**Table 2:** Differentially expressed genes in the MAPT 17q.21.31 locus

Gene	Cerebellum						Temporal cortex					
	logFC	Average Expression	t-statistic	*p* value	FDR adjusted *p* value	Beta	LogFC	Average Expression	t-statistic	*p* value	FDR adjusted *p* value	Beta
*MAPT*	0.279	8.898	6.698	3.53 × 10^−10^	6.91 × 10^−8^	12.783	0.159	8.789	2.537	0.012	0.064	− 3.356
*KANSL1*	0.268	6.754	5.615	8.67 × 10^−8^	2.56 × 10^−6^	7.432	0.182	5.533	3.834	1.82 × 10^−4^	4.09 × 10^−3^	0.297
*NSF*	− 0.182	7.471	− 2.409	0.017	0.039	− 4.010	− 0.115	7.830	− 0.836	0.404	0.614	− 6.166
*PLEKHM1*	0.406	5.895	6.682	3.86 × 10^−10^	7.33 × 10^−8^	12.679	0.240	4.916	4.531	1.15 × 10^−5^	6.93 × 10^−4^	2.892
*ARL17A*	0.023	5.007	0.264	0.792	0.848	− 6.775	− 0.014	3.887	− 0.151	0.880	0.940	− 6.545
*LRRC37A*	− 0.576	3.113	− 3.951	1.17 × 10^−4^	6.28 × 10^−4^	0.797	− 0.606	2.312	− 3.474	6.63 × 10^−4^	9.24 × 10^−4^	− 0.646
*ARHGAP27*	− 0.195	3.869	− 2.379	0.019	0.041	− 3.911	− 0.242	3.537	− 2.688	7.96 × 10^−3^	0.048	− 3.024
*CRHR1*	− 0.141	4.523	− 1.108	0.270	0.370	− 6.151	− 0.021	2.887	− 0.143	0.887	0.944	− 6.381
*ARL17B*	0.280	2.843	3.042	2.76 × 10^−3^	8.50 × 10^−3^	− 2.066	0.099	1.963	0.988	0.325	0.537	− 5.764

*FC* Fold change

**Fig. 3:**
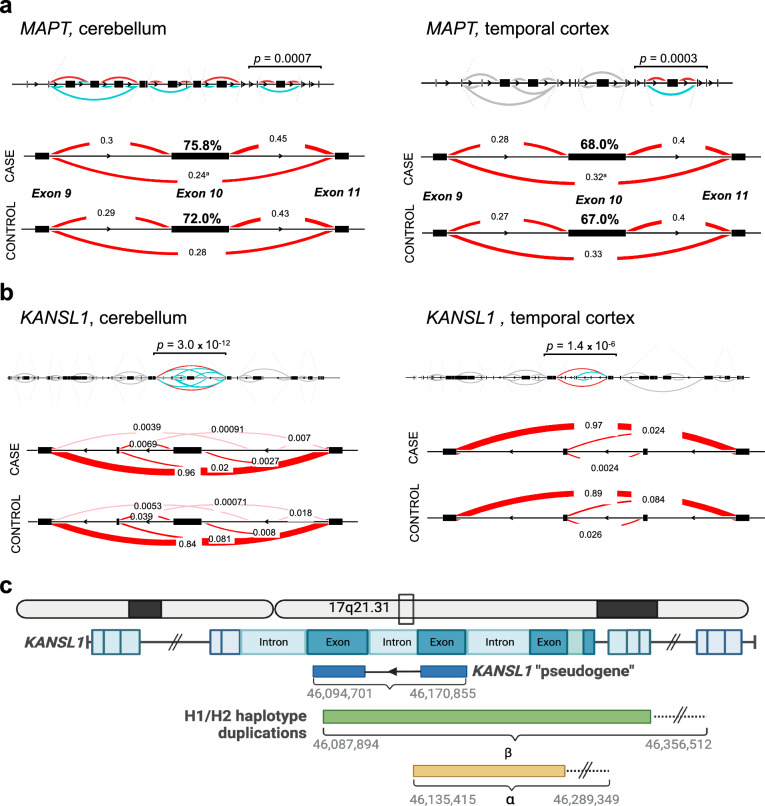
Differential alternative pre-mRNA splicing of *MAPT* and *KANSL1* in PSP cases compared to controls. **a** Full *MAPT* gene with differential inclusion of exon 10 in both brain regions for PSP cases compared to controls. (FDR adj. *p* = 0.0007 in cerebellum, FDR adj.* p* = 0.0003 in temporal cortex). Red lines represent introns annoted in GENCODE, pink lines are novel introns. Each number corresponding to a line represents the relative junction usage within the cluster. **b** Full *KANSL1* gene with differential inclusion of an intron–exon junction in both brain regions (FDR adj. *p* = 3.0 × 10^−13^ in cerebellum, FDR adj. *p* = 0.000001 in temporal cortex). Hg38 Chr17: 46,094,701–46,170,855. **c** Schematic of H1/H2 haplotype breakpoints occurring within the *KANSL1* gene, with potential *KANSL1* pseudogene overlaid

Next, we employed LeafCutter to assess differential intron excision. In total, we found differential detection of intron–exon junctions in 7214 locations in the cortex and 18,802 in the cerebellum between PSP cases and controls. At the 17q21.31 locus, we detected significant differential detection of intron–exon junctions in *MAPT* and *KANSL1* (Fig. 3a, b) as well as *ARHGAP27, LRRC37A, NSF,* and *ARL17A* (Supplemental Fig. 3) in both brain regions. The differences in intron–exon junction detection in *MAPT* at exon 10 indicate greater levels of 4R tau mRNA in the PSP cases compared to controls. Additionally, there was differential detection of intron–exon junctions at *KANSL1* in both brain regions. The *KANSL1* transcript start and end points contain breakpoints of known duplications associated with the H1/H2 haplotypes, termed α and β duplication regions [19, 23, 24]. Thus, the differences in *KANSL1* exon utilization may reflect the presence of a transcribed “pseudogene” [25].

**Fig. 4:**
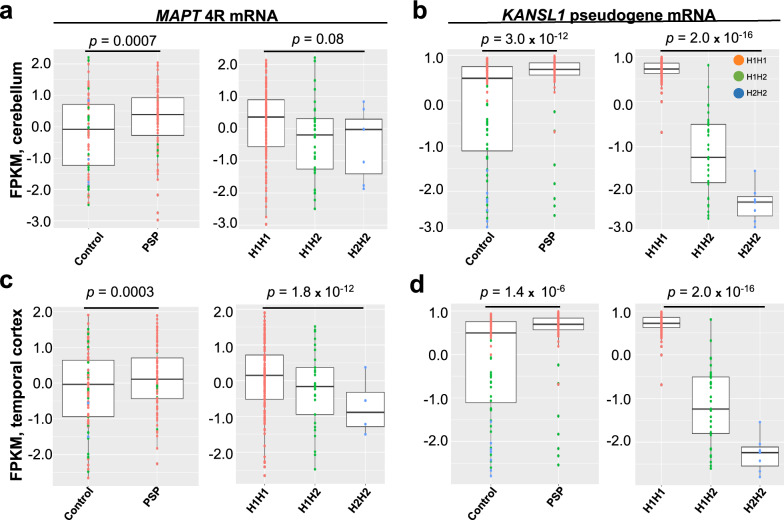
17q21.31 H1 haplotype and progressive supranuclear palsy are both associated with increased 4R tau mRNA and *KANSL1* mRNA isoform expression. Patient haplotype information was determined using the SNP tag at rs1800547. H1 homozygous individuals are shown in orange (n = 77 PSP, n = 44 controls), H1/H2 heterozygous in green (n = 7 PSP, n = 22 controls), and H2 homozygous in blue (n = 0 PSP, n = 4 controls). *q* values are FDR-adjusted p values and were used to determine significance between cases and controls for each isoform. For haplotype-associated isoform and pseudogene expression analysis, a one-way ANOVA was performed

Finally, to assess the underlying basis of our observed differences in *MAPT* and *KANSL1* in PSP, we examined whether the intron–exon junction differences we observed correlate with *MAPT* haplotype status (Fig. 4). Samples were stratified by haplotype status into H1 homozygotes (*n* = 77 PSP, *n* = 44 controls), H1/H2 heterozygotes (*n* = 7 PSP, *n* = 22 controls), and H2 homozygotes (*n* = 0 PSP, *n* = 4 controls). *MAPT* exon 10 inclusion was significantly associated with haplotype in the temporal cortex but not in the cerebellum (*p* = 1.8 × 10^−12^, *p* = 0.08 respectively) whereas the *KANSL1* isoform was significantly associated with haplotype in both brain regions (*p* = 1.0 × 10^−16^, *p* = 2.0 × 10^−16^ respectively). When stratifying by status, haplotype is significantly associated with 4R *MAPT* and *KANSL1* isoform for both cases and controls in the cerebellum. In the temporal cortex, the *KANSL1* isoform is significantly associated with haplotype but 4R *MAPT* is not (Supplemental Fig. 5).

## Discussion

The strong association of PSP and other tauopathies with common variation in the 17q21.31 locus in the absence of a *MAPT* coding region mutation favors genetic regulatory mechanisms as a causal driver [15–17, 37, 51–60]. The hypothesis that alterations in tau mRNA level drive pathology is supported by extensive data, including the observation that duplication of genomic regions including *MAPT*, which increases tau mRNA levels in a dose dependent fashion, is sufficient to cause tauopathy [61]. Here, we tested the hypothesis that increased levels of *MAPT* mRNA and/or changes in alternative *MAPT* pre-mRNA splicing are drivers of disease in PSP by re-examining a bulk RNA sequencing dataset derived from post-mortem human brain tissues using a rigorous computational transcriptomic pipeline. A key component of this analysis was the LeafCutter algorithm that allowed us to assess mRNA expression at relevant splice junctions. The most important finding was that there are increased levels of both total and 4R tau mRNA in the brain tissue of patients with autopsy-confirmed PSP in the cerebellum but not the temporal cortex, indicating *MAPT-*specific expression changes in a brain region more vulnerable to degeneration in this disease. These findings suggest a causal mechanism whereby toxic tau accumulation in PSP arises secondarily to increased total tau and 4R tau synthesis.

We also identified a *KANSL1* transcript strongly associated with PSP and the H1 haplotype, which arises from transcription of a *KANSL1* pseudogene resulting from the H1-haplotype-associated β duplication. Whether this pseudogene is translated and plays a functional role in PSP pathogenesis remains an open question. *KANSL1* is a critical gene in neurological development and function, as highlighted by its involvement in Koolen de Vries syndrome (KdVS) [62–65]. KdVS, caused by deletions or mutations in the *KANSL1* gene, is characterized by developmental delays, intellectual disability, and distinct facial features, underscoring the importance of *KANSL1* in brain development and cognitive function [66, 67]. The association of *KANSL1* disruptions with such a profound developmental disorder emphasizes the potential impact of *KANSL1*-related mechanisms in neurodegenerative diseases like PSP. The gene’s role in KdVS illustrates its broad influence on neurodevelopment, which could provide insights into how its dysregulation may contribute to tauopathies. Understanding the specific contributions of the *KANSL1* pseudogene in PSP could, therefore, reveal critical pathways and targets for therapeutic intervention, further underscoring the significant role of *KANSL1* in neuronal function.

There are limitations to this study that influence the interpretation of our results. First, the dataset had only the temporal cortex and cerebellum, but PSP involves numerous brain regions, including the subthalamic nucleus, globus pallidus, and substantia nigra which were not sampled here [28, 68]. Another limitation of the study is the dataset was derived from bulk-tissue analysis. RNA-seq studies suffer from the inability to resolve cellular heterogeneity. Using estimates based on canonical markers of neurons and glia, we found that there were significant differences in the cellular composition of our tissue. We were unable to control for these differences due to lack of ability to validate the proportions in the original tissue. Studies with greater cell-type specificity are needed to elucidate cell-specific gene expression changes. To this point, recently published single-cell RNA-seq datasets have emerged that implicate specific cell types in the observed differences in *MAPT* expression [31, 69, 70], and further studies are warranted. Additionally, advanced isoform-specific in situ hybridization techniques have allowed for the ability to validate RNA sequencing in post-mortem human tissues, although challenges have arisen in validating the specificity of these probes which would greatly strengthen this study as validation. Additionally, we used differential splicing analysis, Leafcutter, a software tool used to detect differences in intron–exon junctions but may not be as specific as long-read sequencing for detecting differences in isoform prevalence [71]. This analysis also should be validated through an additional splicing detection method. Lastly, we did not look at 17q21.31 sub-haplotypes (i.e. alpha, beta, gamma) and other structural variations that may play a role in gene expression changes. Given these limitations we are planning to complete these experiments as follow up studies.

Here, we provided further evidence supporting the argument that in regions vulnerable to disease, increased synthesis of tau, rather than dysregulated tau degradation, is a critical pathogenic aspect of abnormal tau proteostasis in PSP. These findings suggest that the reduction of tau synthesis may be of utility in treating tauopathy. There are currently several therapeutics targeting tau in phase 1, 2, and 3 clinical trials including monoclonal antibodies, tau-aggregation inhibitors, immunotherapy, and mRNA-based therapies [3, 71–75]. The nuanced understanding of the relationship between tau isoform expression, disease pathology and common genetic variation is integral for development of precision medicine approaches in tauopathy. As such, future research should continue to explore the therapeutic efficacy of these approaches, potentially combining them with other strategies aimed at mitigating neurodegeneration. Overall, the insights gained from this study highlight the importance of ongoing research into the molecular underpinnings of tauopathies and reinforce the promise of targeted therapeutic interventions in the context of rebalancing tau isoform levels.

### Supplementary Information

Additional file 1

Additional file 2

## Data Availability

All the data used in this study was taken from publicly available sources detailed in the methods section of the manuscript and the final processed data is available in the supplementary material.
