# Supplementary material for: MAPT haplotype-associated transcriptomic changes in progressive supranuclear palsy
Source: Acta Neuropathol Commun. 2024 Aug 17;12:135. doi: 10.1186/s40478-024-01839-3 (PMC11330133; doi:10.1186/s40478-024-01839-3)
Supplement: Supplementary file 2 — Additional file 2 [file 40478_2024_1839_MOESM2_ESM.pdf]

Supplemental Figure 1

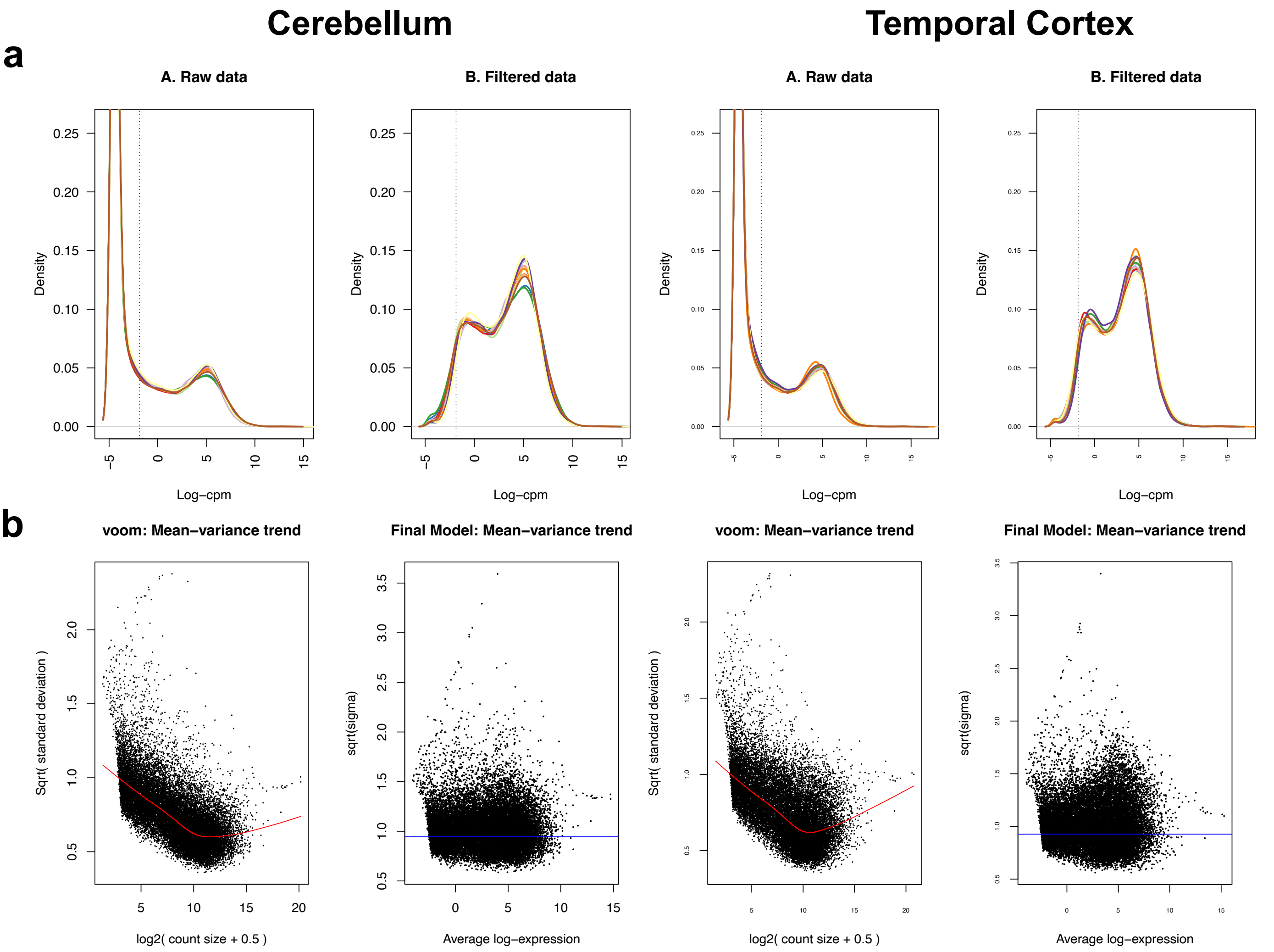

Supplemental Figure 1. Limma R Package removed lowly expressed reads and normalized expression based on mean-variance trends. a. Lowly expressed reads were filtered from the cerebellum (left) and temporal cortex (right). Data is shown before and after filtering. Each color represents a different sample. **b.** Voom was used to normalize reads based on mean-variance trend.

Supplemental figure 2

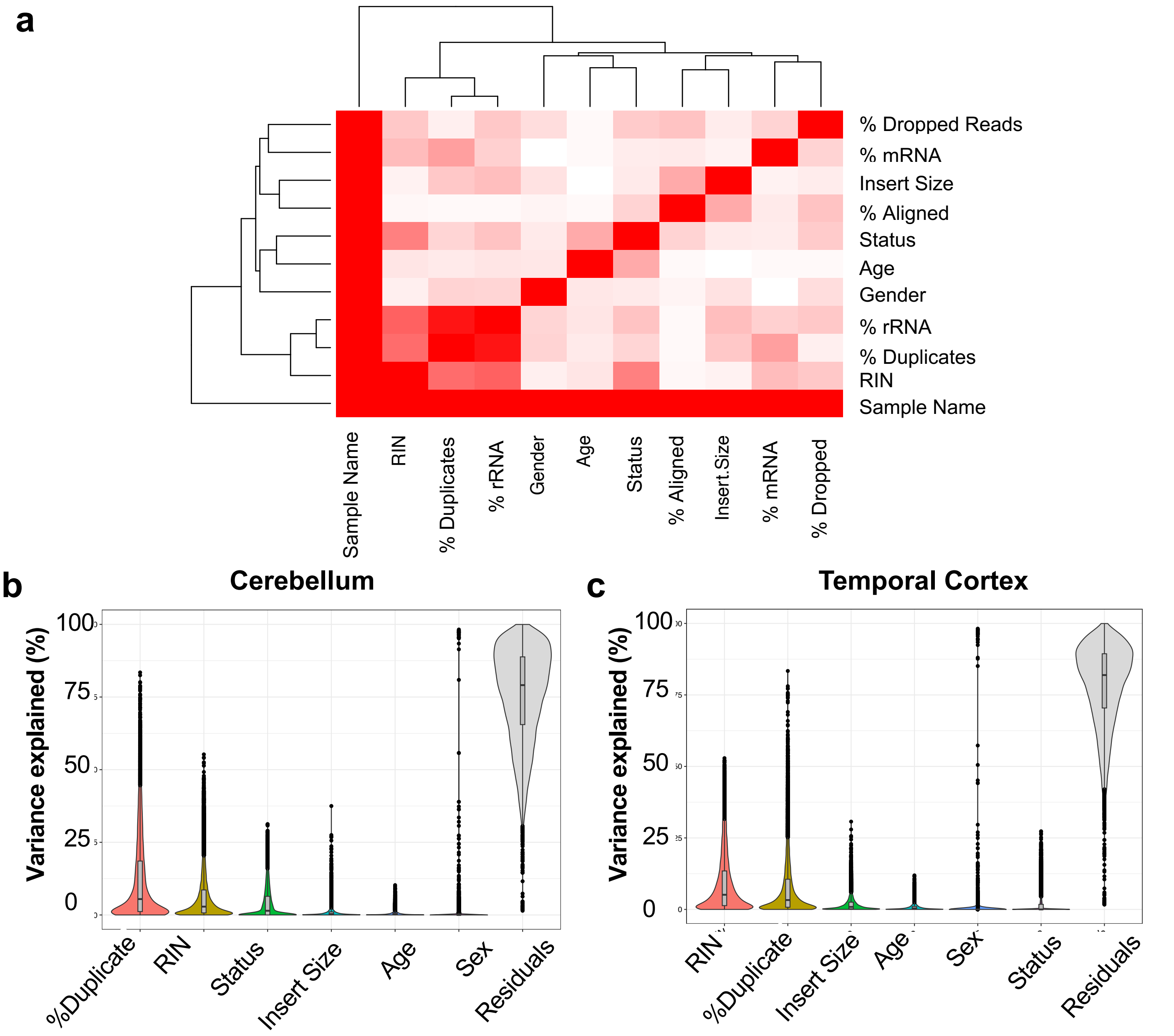

Supplemental Figure 2. Picard technical and sequencing variables were correlated using variancePartition. a. Variables were correlated using variancePartition to select independent covariates for downstream analysis and exclude redundant variables. RIN score, mean insert size, percent duplicate reads, age, gender, and status were selected as final covariates. Their relative contribution to the gene expression variance in the samples are depicted in **b** (cerebellum) and **c** (temporal cortex). RIN, RNA integrity number. Covariates were chosen to maintain approximately 75% variance explained by residuals

Supplemental Fig 3

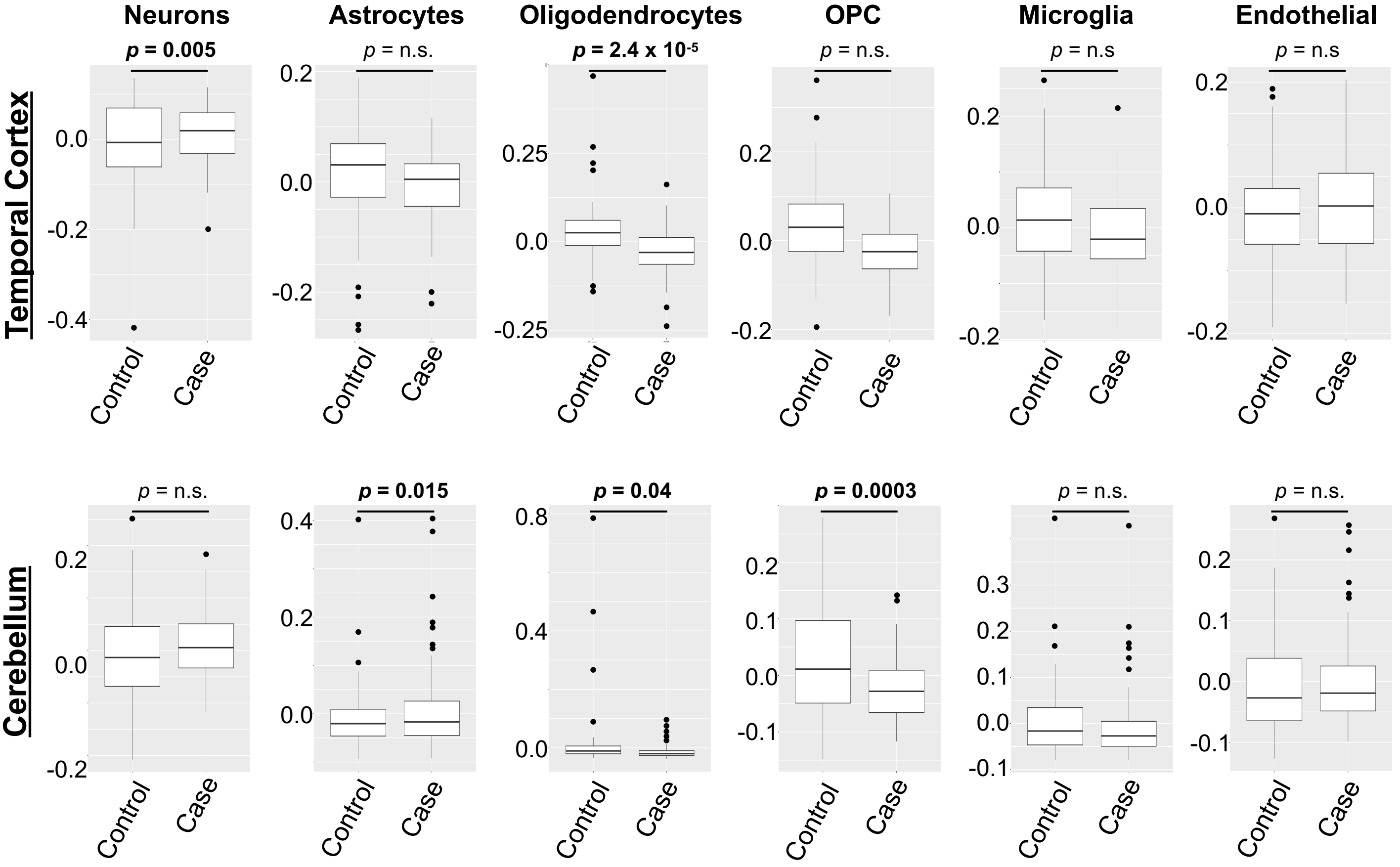

Supplemental Figure 3. BRETIGEA cell type deconvolution results. Cell type proportions were estimated based on canonical markers of gene expression. Relative proportion of cells in each sample is demonstrated in each box plot. There was a significant difference in the relative proportion of neurons and oligodendrocytes in the temporal cortex. There was a significant difference in the relative proportion of astrocytes, oligodendrocytes and OPCs in the cerebellum. OPC, Oligodendrocyte progenitor cell

Supplemental Fig 4

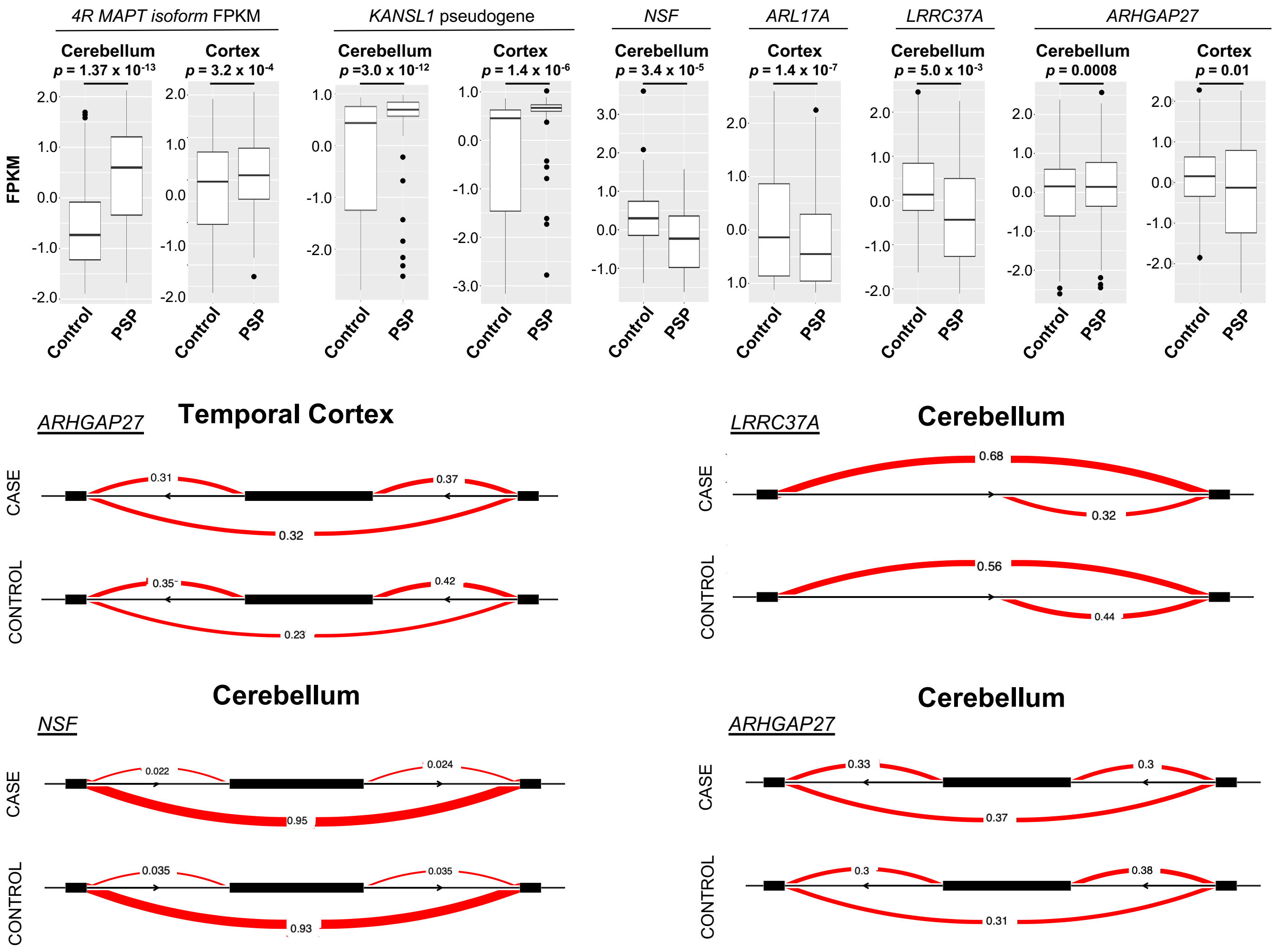

Supplemental Figure 4. Differential splicing of other genes at the 17q21.31 locus. *MAPT* and *KANSL1* intron-exon junction differences displayed in boxplots shown in detail in Fig 3a and b. NSF, ARL17A, LRRC37A, and ARHGAP27 also showed differential splicing. ARL17a sashimi plot is not shown due to high number of breakpoints as a result of haplotype inversion.

Supplemental Fig 5

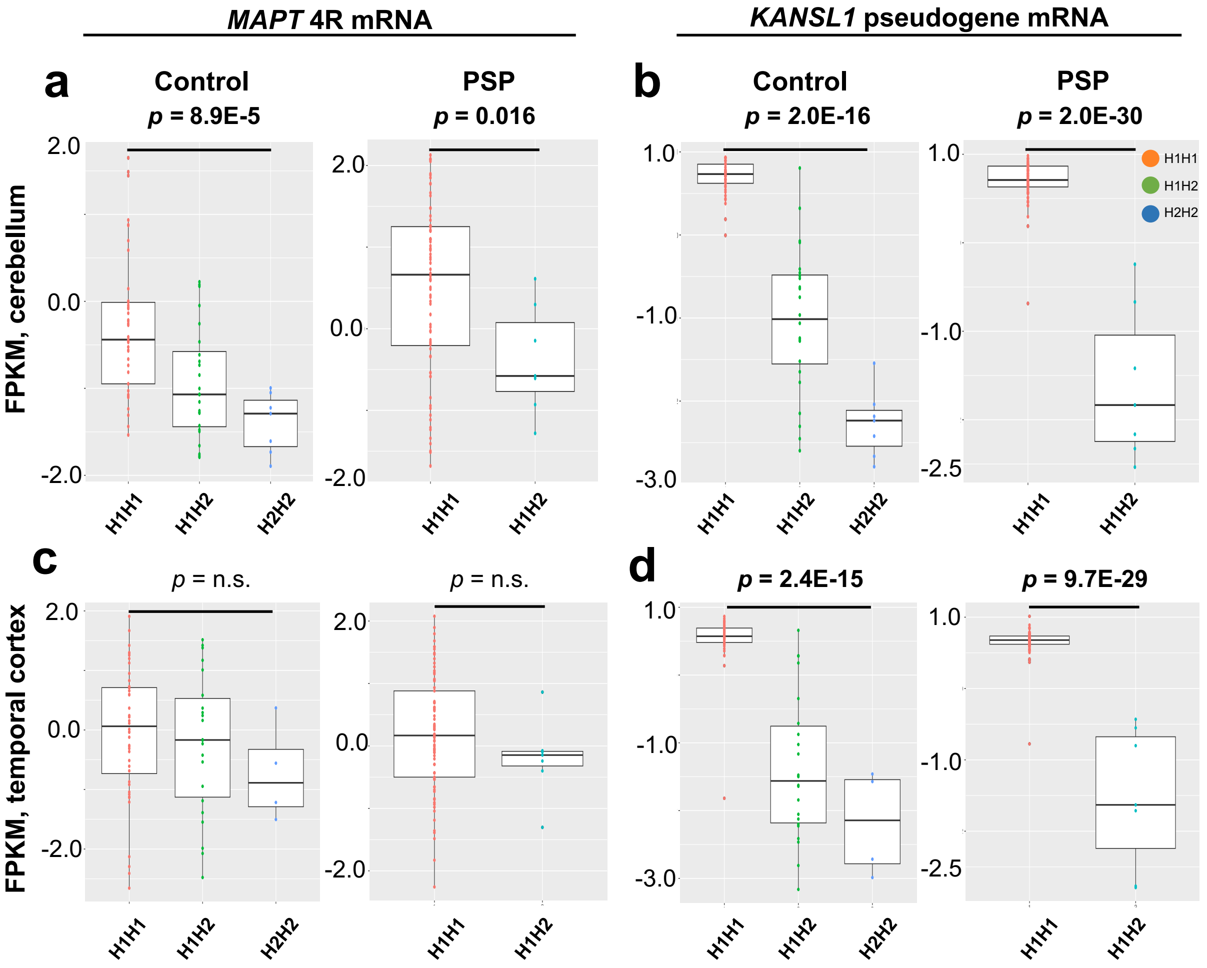

Supplemental Figure 5. 4R tau mRNA and *KANSL1* mRNA isoform expression by disease and haplotype status. Patient haplotype information was determined using the SNP tag at rs1800547. H1 homozygous individuals are shown in orange (n = 77 PSP, n = 44 controls), H1/H2 heterozygous in green (n = 7 PSP, n = 22 controls), and H2 homozygous in blue (n = 0 PSP, n = 4 controls). For haplotype-associated isoform and pseudogene expression analysis, a one-way ANOVA was performed. T test was performed for PSP cases since there were only 2 comparison groups with 0 PSP patients in the H2H2 group.
